# Signatures of Extreme Events in Cumulative Entropic Spectrum

**DOI:** 10.3390/e27040410

**Published:** 2025-04-10

**Authors:** Ewa A. Drzazga-Szczȩśniak, Adam Z. Kaczmarek, Marta Kielak, Shivam Gupta, Jakub T. Gnyp, Katarzyna Pluta, Zygmunt Ba̧k, Piotr Szczepanik, Dominik Szczȩśniak

**Affiliations:** 1 Department of Physics, Faculty of Production Engineering and Materials Technology, Czestochowa University of Technology, 19 Armii Krajowej Ave., 42200 Czestochowa, Poland; ewa.drzazga@pcz.pl; 2 Institute of Physics, Faculty of Science and Technology, Jan Dlugosz University in Czestochowa, 13/15 Armii Krajowej Ave., 42200 Czestochowa, Poland; adam.kaczmarek@doktorant.ujd.edu.pl (A.Z.K.); m.kielak.m@gmail.com (M.K.); katarzyna.w.pluta@gmail.com (K.P.); z.bak@ujd.edu.pl (Z.B.); 3 EntropyX Labs Pvt. Ltd., Ghaziabad 201010, Uttar Pradesh, India; shivam@entropyxlabs.com; 4 Condensed Matter Spectroscopy Division, Faculty of Mathematics, Physics and Informatics, University of Gdansk, 57 Wita Stwosza Str., 80308 Gdansk, Poland; jakub.gnyp@ug.edu.pl; 5 Institute of Pricing and Market Analysis, Analitico, 49/8 Krolewska Str., 47400 Raciborz, Poland; pszczepanik@instytut-analitico.pl

**Keywords:** entropy, information theory, econophysics, extreme events, time series, data science

## Abstract

In this study, the cumulative effect of the empirical probability distribution of a random variable is identified as a factor that amplifies the occurrence of extreme events in datasets. To quantify this observation, a corresponding information measure is introduced, drawing upon Shannon entropy for joint probabilities. The proposed approach is validated using selected market data as case studies, encompassing various instances of extreme events. In particular, the results indicate that the introduced cumulative measure exhibits distinctive signatures of such events, even when the data are relatively noisy. These findings highlight the potential of the discussed concept for developing a new class of related indicators or classifiers.

## 1. Introduction

The ability to differentiate between volatility inherent to the given data and introduced by a temporal dependence on external variables is necessary for efficient modeling. This is particularly important in the case of significant anomalies, or extreme events, and their impact on the overall distribution of the gathered data. For this purpose, an analytical tool with a solid theoretical foundation and straightforward implementation is required. To obtain such a tool, it is essential to understand what constitutes an extreme event and what are its characteristics in an ex post analysis. Extreme events are typically represented by imbalanced data in a time series, which occur irregularly and introduce either extremely low or high values [1,2]. This implies knowledge of an expected magnitude of the data, with all that lay beyond labeled as extreme. One way to express this is by stating that values resulting from extreme events deviate by more than several standard deviations. As such, extreme events span various domains, from science and technology to social studies, and may include sudden outbreaks of devastating infectious diseases, solar flares, extreme weather conditions, or financial crises [2,3,4,5].

The unexpected and complex characteristics of extreme events introduce significant challenges in their theory and modeling. In particular, since all these events often result from strong non-linear interactions across various lengths and time scales, they render conventional perturbative methods less effective [6]. Unfortunately, artificial intelligence has yet to come to the rescue. Although, there have been attempts to mitigate the discussed problems via machine learning (both classical and quantum), there are not always enough data or computational power to perform such simulations [7,8]. Finally, it is important to note that these extreme events contribute to the tails of probabilistic distributions, having minimal effect on mean values but significantly impacting volatility and variance. Interestingly, this opens a promising avenue, since one way to analyze volatility is through the measure known as entropy [9,10,11,12,13], an analytical concept that also underlines the information theory [14]. In this sense, entropy estimates the uncertainty and randomness of a dataset, enabling the discussion of its related fluctuations, distributions, and patterns [9,15,16,17,18,19,20,21]. As a result, entropy constitutes a potentially relevant framework for discussing the impact of sudden or extreme events across various fields. This includes problems such as abrupt changes in the volatility of economic data [11,13], the detection of earthquakes [22], sudden events in systems management [23], or climate changes [24]. Still, caution is advised when applying entropy in this manner, as it may not always be sufficient to solely capture all the aspects of extreme processes [25].

In this context, it is argued that entropy may serve as a promising, albeit specific, indicator of extreme events, according to its inherent nature and previous studies [13,25]. Here, this claim is formally justified by the following argument. It is known that entropy increases as the uncertainty (volatility) of the data rises, meaning that the extreme events should result in heightened entropy [13,21]. Hence, a simple cumulative process can be considered to further magnify this aspect, leading to a characteristic pattern in the entropic spectrum. This process relates directly to the evolution of empirical probability distribution. As more data points are taken into account, the observed probability distribution (empirical distribution) gradually converges to the true probability distribution, in accordance with the law of large numbers [26] and the Glivenko–Cantelli theorem [27,28,29]. Note that there is no universal threshold for this convergence; however, the key point here is that the process occurs gradually along with the increasing data size. Under this assumption, entropy may decrease with a balanced dataset, signifying a reduction in uncertainty and the dominance of a few stable outcomes. By systematically comparing the cumulative distributions obtained in such a process with the expected fat-tailed distribution of the extreme event data, the desired amplification of the latter can be effectively achieved. This approach may be particularly significant in terms of a retrospective analysis, tracing back from an extreme event while cumulatively incorporating data. As a result, it should be possible to develop the necessary indicators or classifiers for sudden changes in data by considering such events as a reference point in time.

This work is organized as follows: Section 2 introduces the methodology and theoretical background based on the concept of entropy. Section 3 explores the properties of the data used in this study and provides a detailed analysis of the extreme event signatures within the entropic spectrum. The manuscript concludes in Section 4, which offers a summary and outlines the future perspectives. The study is supplemented by Appendix A, summarizing the statistical analysis of the data.

## 2. Methodology

To quantify the cumulative effect, we recall the conventional discrete Shannon entropy, given by the following [14]:

(1) $$\begin{array}{c} H=-\sum_{i=1}^{n}p\left(x_{i}\right)\ln p(x_{i}), \end{array}$$

The above-mentioned equation is employed for a total of *n* outcomes, which are interpreted here as the “intervals” (also called “bins” or “classes”) within the histogram corresponding to the probability distribution. To ensure consistency across the analysis, the value of *n* is assumed after the Vellman formula, which is optimal given the population and variability of the discussed datasets [30]. We expand on Equation (1) as follows:

(2) $$\begin{array}{c} p\left(x_{i}\right)=\left(x_{i+1}-x_{i}\right)f\left(x_{i+1}\right), \end{array}$$

where the probability of value $x_{i}$ occurring for a discrete random variable of interest is represented. The probability written here is in the Riemann approximation, where $x_{i}$ and ($x_{i+1}$) are the the left and right width endpoints of an interval, whereas $f(x_{i+1})$ stands for the corresponding height. As such, Equation (1) measures the information content of the data in nats (meaning the base of the logarithm in Equation (1) is *e*), accounting for the probability distribution across all possible states.

Now, let us consider a dataset that contains information about an extreme event, along with some preceding data. This dataset is constructed in a way that it can be divided into equally sized parts or blocks, representing subsets comprising an equal number of data points. The aim is that only one such block will consist of data corresponding to the extreme event. As a result, it can be qualitatively argued that the data in each block exhibit a relatively similar probability distribution, except for the subset corresponding to the extreme event. Based on the “most biased distribution principle”, which has been applied in some stochastic processes [21], the subsets unrelated to the extreme event will manifest a bias toward a few stable outcomes, which will be further amplified when their corresponding probability distributions are combined. This observation can be quantified by introducing the following cumulative entropy:

(3) $$\begin{array}{c} H_{m}=-\sum_{i=1}^{n}p\left(x_{i,0},x_{i,1},\ldots ,x_{i,m}\right)\ln p(x_{i,0},x_{i,1},\ldots ,x_{i,m}), \end{array}$$

which is formally a joint entropy for *m* discrete random variables, where $p(x_{i,0},x_{i,1},\ldots ,x_{i,m})$ represents the joint probability that captures the likelihood of the simultaneous occurrence of $x_{i,0},x_{i,1}\ldots ,x_{i,m}$ values [26]. This probability is computed in a straightforward manner, following the same approach as in Equation (2). In this framework, when m=0, Equation (3) converges to Equation (1) and yields entropy for the subset with the fewest data points. This is considered as a reference entropy value. On the other hand, as *m* increases, more data and corresponding information are encompassed within the cumulative entropy. Such a process increases the discrepancy between the cumulative entropy for higher non-zero *m* and the case when m=0, that is, the entropy value corresponding to the extreme event can be magnified for better detection, as initially desired.

Note that, by definition, $H_{m}$ is a non-negative and sub-additive quantity, inheriting these characteristics from Equation (1). In detail, non-negativity occurs due to the fact that $0\leq p(x_{i,0},x_{i,1},\ldots ,x_{i,m})\leq 1$, meaning that $\ln p(x_{i,0},x_{i,1},\ldots ,x_{i,m})\leq 0$ for probabilities in (0,1]. Hence, since $p\left(x_{i,0},x_{i,1},\ldots ,x_{i,m}\right)\ln p(x_{i,0},x_{i,1},\ldots ,x_{i,m})$ is always non-negative for 0<p≤1, the summation in Equation (3) remains non-negative. On the other hand, $H_{m}$ is sub-additive because the considered discrete variables are dependent. In such a situation, again by definition, $H_{m}\leq H_{m-1}-\sum_{i=1}^{n}p\left(x_{i,m}\right)\ln p(x_{i,m})$. Thus, the cumulative entropy does not grow faster than the sum of individual entropies, proving sub-additivity. As such, it is also important to note that Equation (3) does not rely on any assumptions about the underlying probability of a dataset; it instead seeks to uncover its intrinsic characteristics through entropy.

## 3. Results and Discussion

To validate the cumulative entropy concept and its underlying rationale, several benchmark datasets are examined. In particular, these consist of market data centered around three key dates, each corresponding to a selected extreme event that occurred in the last decade, as follows:

- 24 June 2016, marking the announcement of the Brexit referendum results [31];
- 16 March 2020, recognized globally as Black Monday, which represents the economic panic due to the COVID-19 pandemic [32];
- 24 February 2022, denoting the beginning of the Russian invasion of Ukraine [13].

All the above-mentioned events are captured in the context of a time series of exchange rates between gold and the U.S. dollar. The total data coverage spans 41 working days, consisting of 30 working days before the event, the day of the extreme event itself, and 10 working days after it. The frequency of each dataset is one data point every half hour. In this manner, the considered datasets are well-suited for the cumulative entropy calculations, as they can be divided into subsets, where only one of them contains data related to the extreme event, while the others consist of data points leading up to it. However, an additional comparative analysis is also possible when the reference value of entropy, as defined within Equation (3), is assumed to correspond to the subset unrelated to the extreme event. In other words, the reference point can be set either before or after the extreme event.

**Figure 1:**
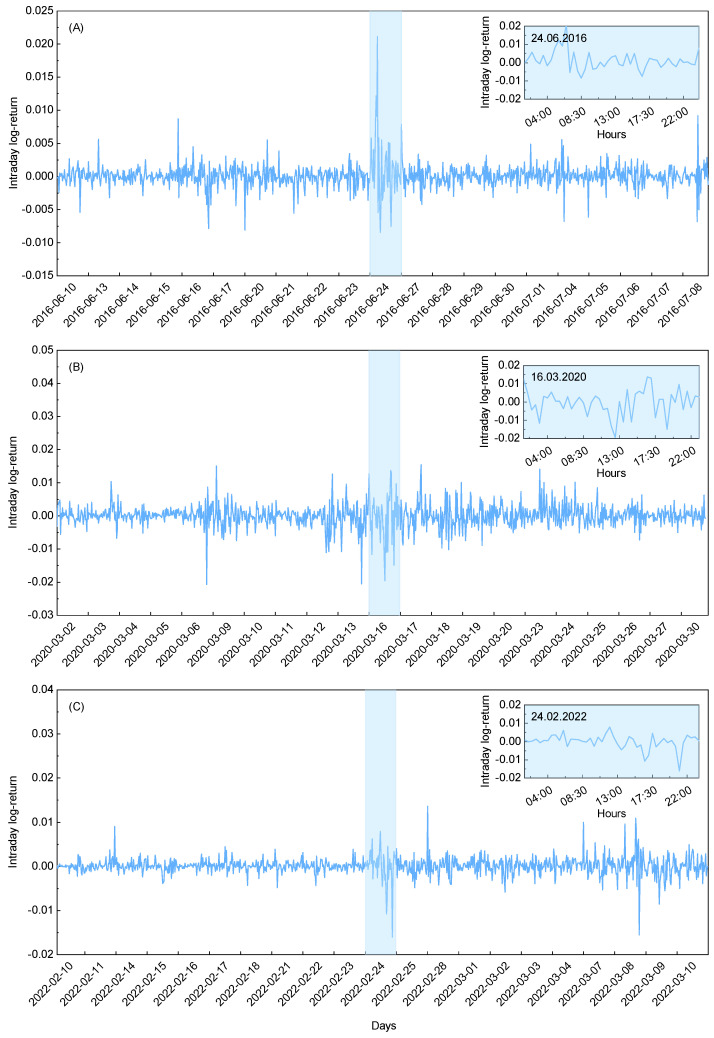
The intraday log-returns for the exchange rates between gold and the U.S. dollar during different time periods. Each subfigure is associated with one of the three extreme events of interest: (**A**) the Brexit referendum results, (**B**) Black Monday due to COVID-19, and (**C**) the Russian invasion of Ukraine. The data range for all the subfigures is restricted to the given extreme event day (blue shaded area) ± 10 days. The central regions are additionally magnified in the insets, with the corresponding dates depicted.

For convenience, information about the time series of interest is encoded via intraday log-returns ($r_{j}$) as follows:

(4) $$r_{j}=\ln\frac{p_{j}}{p_{j-1}}\approx \frac{p_{j}-p_{j-1}}{p_{j-1}},$$

where $p_{j}$ ($p_{j-1}$) is the closing price of an asset in the *j*-th (j−1-th) half-hour interval. Such log-returns serve as a stationary time series representation of the price changes, capturing the relative magnitude of intraday fluctuations. The graphical representation of the intraday log-returns for the exchange rates between gold and the U.S. dollar is presented in Figure 1A–C, across three different time periods. The presented data are based on one of the three above-mentioned extreme events, namely the Brexit referendum results (see Figure 1A), Black Monday due to COVID-19 (see Figure 1B), and the Russian invasion of Ukraine (see Figure 1C). For clarity and transparency, the data range is restricted to the extreme event day ± 10 days. The extreme event day is additionally marked by the blue shaded area and magnified in the inset for further details. It can be seen that the depicted returns qualitatively exhibit the expected increase in turbulence within the blue shaded area, as evidenced by the strong deviations from equilibrium. This effect is particularly pronounced in Figure 1A,C, which illustrate data behavior for the first and third event, respectively. Therein, the transient deviations are nearly four times the equilibrium value. In comparison, the second midterm event is much noisier across the entire time range, constituting an interesting case study when the event of interest is less evident. Subsequently, this example extends the presented analysis to the cases where event detection is more difficult, allowing us to benchmark the cumulative entropy concept in complex scenarios. For further details on the considered datasets, please refer to Appendix A, where a summary of the statistical analyses is provided.

**Figure 2:**
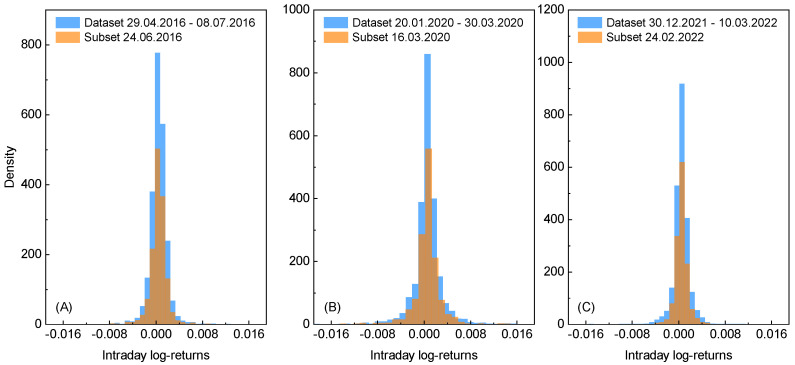
The discrete probability distributions of log-returns for the exchange rates between gold and the U.S. dollar, corresponding to different time periods. Each subfigure is associated with one of the three extreme events of interest: (**A**) the Brexit referendum results, (**B**) the Black Monday due to COVID-19 and (**C**) the Russian invasion of Ukraine. The subfigures depict results for total datasets spanning range of 41 working days (blue color) and their subsets related to the given extreme event day only (orange color). The corresponding time periods and dates are depicted.

However, in the present paper, it is argued that an extreme event is not only reflected in the spectrum of intraday log-returns but can be also observed in the corresponding empirical probability distribution. In Figure 2A–C, such approximate discrete distributions of the intraday log-returns are presented for each total dataset (blue color) considered and for their corresponding subsets that refer to the day when a given extreme event occurs (orange color). Upon analyzing these results, crucial observations can be made that confirm the earlier arguments. The total data are relatively more dispersed than the data distribution for the extreme event day. That means information contained within the latter dataset is less ordered and the corresponding outcome is more uncertain. This clearly shows that the data for the extreme event day incorporate some randomness into the associated total dataset. In other words, as more data are introduced into a dataset, the discrepancy between the resulting distribution and the distribution for a single day subset increases. Since this cumulative effect is directly related to the information content within the data, it can be quantified using entropy.

**Figure 3:**
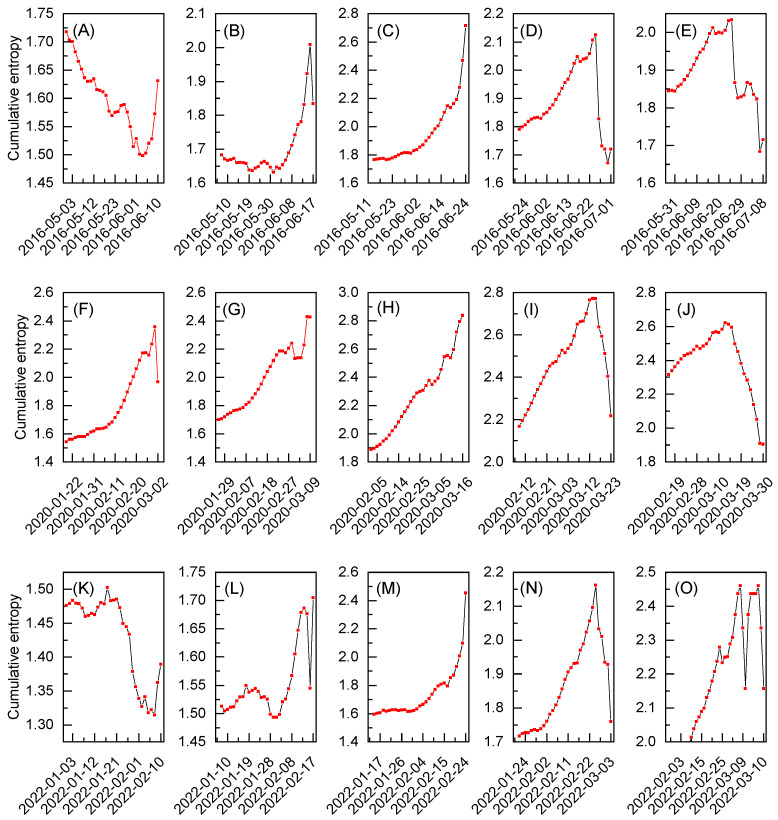
The cumulative entropy for the exchange rates between gold and the U.S. dollar during various periods of time. Each subfigure corresponds to a different time window within one of the datasets centered around a specific extreme event of interest: (**A**–**E**) the Brexit referendum results, (**F**–**J**) Black Monday due to COVID-19, and (**K**–**O**) the Russian invasion of Ukraine. The data range for each subfigure is restricted to 30 working days.

In Figure 3A–O, the behavior of cumulative entropy for the exchange rates between gold and the U.S. dollar during various periods of time is presented. Each subfigure corresponds to a different time window within one of the datasets centered on a specific extreme event of interest, namely the Brexit referendum results (see Figure 3A–E), Black Monday due to COVID-19 (see Figure 3F–J), and the Russian invasion of Ukraine (see Figure 3K–O). The time windows are constructed in accordance with Equation (3), assuming the last point as a reference entropy value. The analysis starts with the time windows ending 10 working days before a given extreme event (see Figure 3A,F,K), before advancing sequentially in 5-working-day increments (see Figure 3B–D,G–I,L–N), ultimately concluding with time windows for the reference points 10 working days after the extreme event (see Figure 3E,J,O). Considering this, the middle column (see Figure 3C,H,M) relates to the cases when each reference entropy value corresponds to one of the extreme event days, and the presented results are expected to exhibit some characteristic patterns. Indeed, the cumulative entropy, as depicted in the middle column, steadily increases and reaches its maximum at a reference point. In the first and third rows (see Figure 3C,M), this increase resembles parabolic behavior and corresponds to the datasets where the intraday log-returns for the extreme event day present well-indicated deviations from the rest of the data (see Figure 1A,C). On the other hand, in the middle row (see Figure 3H), the underlying data are noisy, and the behavior of the results obtained for cumulative entropy is more linear. Still, this increase is continuous, without any substantial drops. As such, all three sets of results, given in Figure 3C,H,M, clearly present somewhat ordered behavior that differentiate them from the cases where the reference point for calculations is assumed several days after or before the extreme event day. To some extent, an exception to this rule is seen in the results presented in Figure 3G, where entropy increases, with only a slight dip around 4 March, reaching nearly its highest value on 9 March. Interestingly, this date is considered yet another Black Monday during the 2020 market crash, although it is expected to correspond to smaller deviations from the equilibrium than the data from 16 March [32]. Furthermore, the downturn observed in the entropic spectrum can be attributed to the behavior of log-returns in Figure 1B, which remained stable from 4 March to early 6 March, before experiencing a sharp decline just before the weekend. Still some resemblance to the patterns presented in Figure 3C,H,M can be observed. The presented results prove that the cumulative entropy may exhibit signatures of interest, even when a dataset is relatively noisy.

The observed behavior also aligns with the earlier arguments, as the entropy value on the day of an extreme event can be amplified by calculating the cumulative data for several days prior to this point. Such a reference entropy value for an extreme event clearly corresponds to the data subset that is most random and provides the most uncertain message. This entropy value is also the highest among all other reference points calculated for the time windows considered within a given dataset (please refer to Appendix A for numerical reference entropy values depicted in Figure 3). However, it is crucial to note that this behavior is possible only when the cumulative entropy is calculated, expanding upon the aforementioned “most biased distribution principle” [21].

In relation to the above, only a few sets of results follow this process and present related patterns. In other words, it seems difficult to obtain the required bias between the reference point and the cumulative data when the former does not correspond to the atypical and significant deviation from the equilibrium that is characteristic for extreme events. However, it is argued that with an appropriate time step or careful real-time analysis, some classifiers or indicators of the extreme events may still be possible to develop. To further verify this, proper statistical validation is required, particularly through a sensitivity analysis. This can be achieved by conducting calculations of the cumulative entropy for a greater number of time windows than those in Figure 3, while simultaneously testing other time window sizes. To provide the most representative results, such an analysis is conducted here for the nosiest of the three considered datasets, which is centered around the extreme event known as Black Monday due to the COVID-19 pandemic.

**Figure 4:**
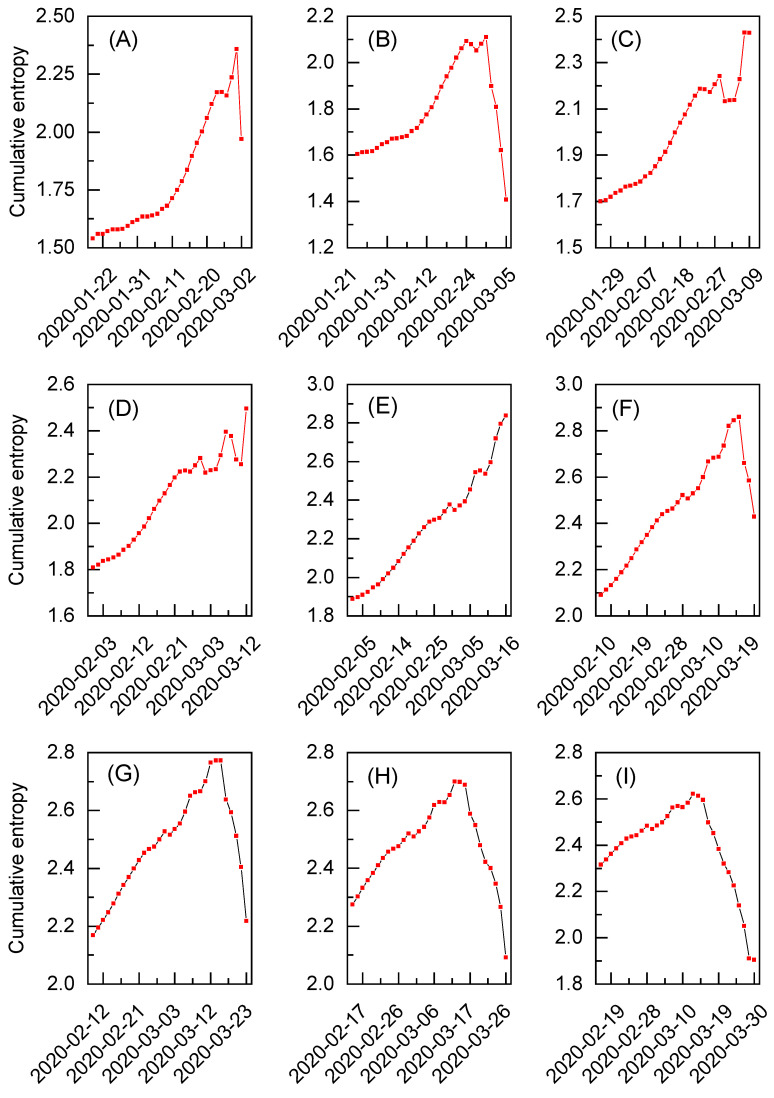
The cumulative entropy for the exchange rates between gold and the U.S. dollar during various time periods. Each subfigure (**A**–**I**) corresponds to a different time window of the dataset based on the extreme event known as Black Monday due to the COVID-19 pandemic. The data range for each subfigure is restricted to 30 working days.

In Figure 4A–I, the cumulative entropy for the exchange rated between gold and the U.S. dollar is depicted for the selected time windows. All the time windows are derived exclusively from the dataset for the Black Monday extreme event. Similarly to Figure 3F–J, these results are obtained for the initial 30 working days, but the number of considered time windows is increased from five to nine (see Appendix A for the corresponding summary statistics). The evident extreme event pattern is visible only in Figure 4E and corresponds to the results initially presented in Figure 3H. However, thanks to the increased number of time windows, it can be observed that the cumulative entropy significantly drops after 16 March for all the subsequent sets of results depicted in Figure 4F–I. It means that no additional extreme events of significant magnitude are detected after this date during the analysis. The situation is different when inspecting time windows preceding the discussed extreme event. Although the results in Figure 4A,B show an initial increase in entropy followed by a decline, potential extreme event patterns can still be observed in Figure 4C,D. The former was already identified in Figure 3G and found to reflect another Black Monday on 9 March [32]. Interestingly, the behavior of cumulative entropy in Figure 4D suggests the presence of an additional extreme event in the analyzed dataset. This pattern can be attributed to the so-called Black Thursday that happened on 12 March [32]. Note, however, that both events present intermediate drops in the entropic spectrum and yield maximum reference entropy values lower than in the case of the results given in Figure 4E (see Appendix A for numerical reference entropy values). As such, the extended analysis not only reveals multiple extreme events in one dataset but also allows us to initially quantify their magnitude via the corresponding reference entropy value.

**Figure 5:**
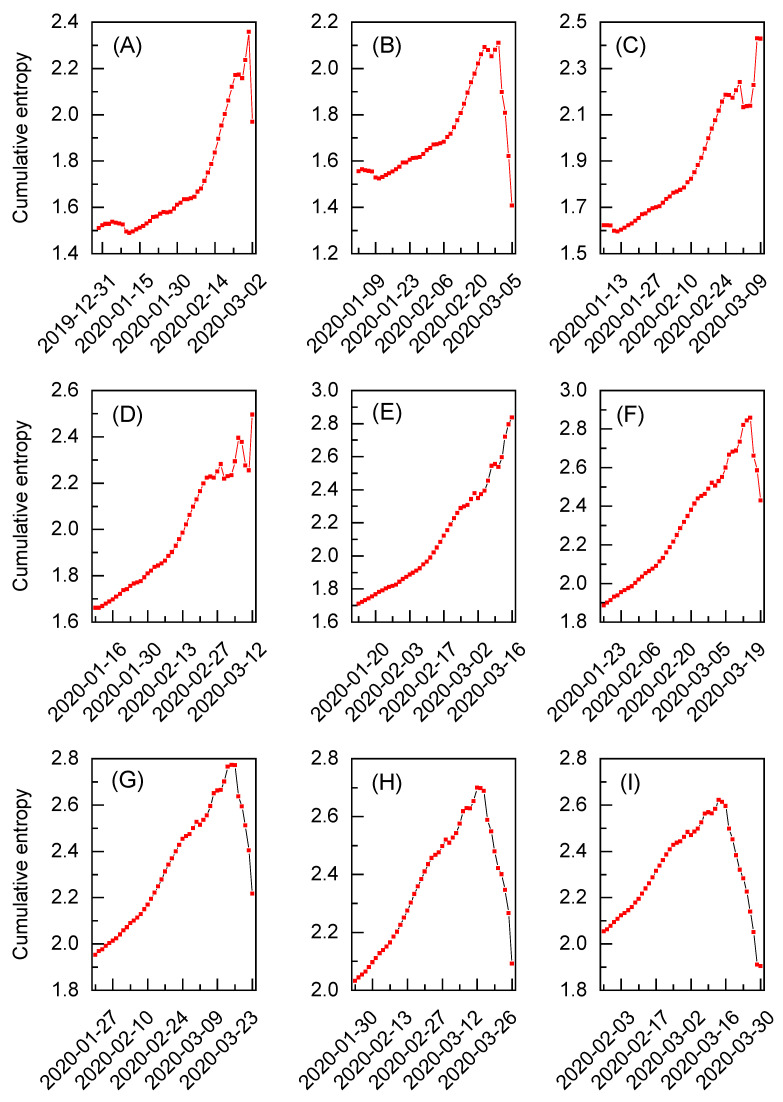
The cumulative entropy for the exchange rates between gold and the U.S. dollar during various time periods. Each subfigure (**A**–**I**) corresponds to a different time window of the dataset centered on the extreme event known as the Black Monday due to the COVID-19 pandemic. The data range for each subfigure is restricted to 45 working days.

These observations are supplemented by the analysis of the cumulative entropy but for larger time windows, now spanning 45 working days. The extended windows are created by adding 15 additional working days to the beginning of each time window employed in Figure 4A–I. Such analogical results are presented in Figure 5A–I and the corresponding summary statistics can be found in Appendix A. For the most part, the behavior of cumulative entropy for the extended windows is similar to that of their shorter counterparts. In general, the results for an additional 15 days increase monotonically without any major disruptions. This may be attributed to the fact that cumulative entropy continues to converge as more data points are incorporated. Only in Figure 5A–C, a few small entropy fluctuations are visible around 9 January; however, these do not influence the general trend. Therefore, no major events are expected to have occurred before those previously identified. Ultimately, the calculated results also validate the sensitivity of the presented method and show that varying the size of the time window does not qualitatively alter the behavior of cumulative entropy.

## 4. Summary and Conlusions

In summary, the analysis in this study was conducted to verify the use of the entropy measure for detecting extreme events in datasets. It was observed that entropy for joint probabilities could be employed in a systematic manner to amplify subsets or blocks that contain information on an extreme event. In particular, these findings were presented for three datasets of choice, containing market data on the exchange rates between gold and the U.S. dollar. Each dataset was associated with one extreme event, namely the announcement of the Brexit referendum results, the global Black Monday due to the COVID-19 pandemic, and the beginning of the Russian invasion of Ukraine, respectively. For all three datasets, the characteristic signatures in the entropic spectrum were obtained, validating the proposed theoretical framework to some extent.

Thus, it can be concluded that the presented method, based on the cumulative entropy, may be beneficial not only for the detection but also for the classification of extreme events in various datasets. It may serve as a primary or supplementary indicator and classifier, building upon the underlying distribution of the considered dataset and the information encapsulated within it. In this manner, the cumulative entropy appears as a universal and comprehensive measure that does not impose any constraints on the corresponding probability distribution, but rather quantifies its underlying and most important features and interdependencies. In this manner, the developed argument and obtained results formalize the earlier preliminary findings on the cumulative entropy concept [13], addressing previously unexplored essential theoretical aspects and providing a corresponding unified framework, along with its initial validation.

The above findings naturally call for further verification using large-scale data. Of particular interest should be noisy datasets with multiple potential extreme events, similar to the one for Black Monday, where the extraction of information on an extreme event is hindered. This would be particularly helpful in extending the statistical validation of the theoretical methods presented here. Another direction may be the implementation of cumulative entropy in real-time techniques, which deal with the short time windows, suggesting an opportunity to use the measures developed here for early warning systems. The presented study also poses questions regarding the potential of using the cumulative entropy or the underlying most biased distribution principle in combination with other techniques, similar to what has been performed recently for the geometric Brownian motion process [21]. To this end, yet another promising directions may be the incorporation of the above-mentioned concepts into machine learning or deep learning techniques, e.g., toward improvements in the predictive capabilities of these methods, as demonstrated by Chowdhury et al. in their study on forecasting extreme events in chaotic systems using long short-term memory (LSTM) networks [33].

## Data Availability

The datasets used in this study are sourced from publicly available repositories. The employed datasets for the exchange rates between gold and the U.S. dollar can be accessed at www.histdata.com, where they are freely available for download. The employed datasets has been accessed on 14 January 2025. In addition, all original contributions and processed data presented in this study are included in the article. For any further inquiries regarding data usage or access, readers may contact the corresponding author.

## Appendix A. Summary Statistics

The appendix provides supplementary data supporting the main analysis. Table A1 presents the summarized statistics of the intraday log-returns for the three datasets covering the extreme events, while Table A2 and Table A3 provide the statistics for the sensitivity analysis dataset.

**Table A1:** The summarized statistics of the intraday log-returns for the three datasets associated with the three extreme events of interest, i.e., announcement of Brexit referendum results on 24 June 2016, Black Monday (which affected the global market) due to the COVID-19 pandemic on 16 March 2020, and the beginning of the Russian invasion of Ukraine on 24 February 2022. The statistics are given for the total period of time (41 working days) considered in the context of each event. The partial statistics are presented for five periods (30 working days) analyzed in terms of the cumulative entropy.

Time Window	Return Count	Mean	Minimum	Maximum	Skewness	Kurtosis	Reference Entropy
Brexit referendum results (24.06.2016)							
29.04.2016–08.07.2016	2399	3.361 × 10^−5^	−0.008445	0.021147	2.04252	28.862991	1.714689
29.04.2016–10.06.2016	1439	5.401 × 10^−6^	−0.007146	0.019459	2.08483	33.766491	1.631333
06.05.2016–17.06.2016	1439	1.281 × 10^−5^	−0.007835	0.019459	1.989836	35.460082	1.83455
13.05.2016–24.06.2016	1439	2.91 × 10^−5^	−0.008445	0.021147	2.831727	39.116341	2.714941
20.05.2016–01.07.2016	1439	4.854 × 10^−5^	−0.008445	0.021147	2.949383	38.295354	1.720074
27.05.2016–08.07.2016	1439	8.143 × 10^−5^	−0.008445	0.021147	2.579468	32.706395	1.714689
Black Monday due to COVID-19 (16.03.2020)							
20.01.2020–30.03.2020	2399	2.07 × 10^−5^	−0.020675	0.015444	−0.510216	11.956491	1.905316
20.01.2020–02.03.2020	1439	1.593 × 10^−6^	−0.01423	0.014090	−0.643282	19.896956	1.969378
27.01.2020–09.03.2020	1439	4.197 × 10^−5^	−0.020675	0.015017	−0.936259	22.677022	2.427632
03.02.2020–16.03.2020	1439	−3.353 × 10^−5^	−0.020675	0.015017	−1.223812	14.9922	2.837796
10.02.2020–23.03.2020	1439	−4.774 × 10^−5^	−0.020675	0.015444	−0.593074	9.164225	2.217087
17.02.2020–30.03.2020	1439	2.24 × 10^−5^	−0.020675	0.015444	−0.455545	7.130588	1.905316
Russian invasion of Ukraine (24.02.2022)							
30.12.2021–10.03.2022	2399	4.437 × 10^−5^	−0.016004	0.013665	−0.567677	19.873723	2.15761
30.12.2021–10.02.2022	1439	9.097 × 10^−7^	−0.009664	0.006961	−0.88388	12.404316	1.389382
06.01.2022–17.02.2022	1439	3.37 × 10^−5^	−0.009664	0.009024	−0.180055	12.974311	1.705062
13.01.2022–24.02.2022	1439	3.029 × 10^−5^	−0.016004	0.009024	−1.642763	26.258791	2.452496
20.01.2022–03.03.2022	1439	3.715 × 10^−5^	−0.016004	0.013666	−0.8126	21.545034	1.759745
27.01.2022–10.03.2022	1439	6.644 × 10^−5^	−0.016004	0.013666	−0.572695	16.616165	2.157609

**Table A2:** The summary statistics of the intraday log-returns for the dataset associated with the extreme event known as Black Monday due to the COVID-19 pandemic, which occurred on 16 March 2020. The statistics are given for the total period of time (50 working days) and for nine periods (30 working days each) analyzed in terms of the cumulative entropy.

Time Window	Return Count	Mean	Minimum	Maximum	Skewness	Kurtosis	Reference Entropy
Black Monday due to COVID-19 (16.03.2020)							
20.01.2020–30.03.2020	2399	2.057 × 10^−5^	−0.020675	0.015444	−0.510216	11.956491	1.905316
20.01.2020–02.03.2020	1439	1.593 × 10^−6^	−0.01423	0.014090	−0.643282	19.896956	1.969378
23.01.2020–05.03.2020	1439	4.949 × 10^−5^	−0.01423	0.01409	−0.358052	16.986339	1.40755
27.01.2020–09.03.2020	1439	1.593 × 10^−5^	−0.014223	0.0141	−0.643282	19.896956	1.96937
30.01.2020–12.03.2020	1439	3.159 × 10^−6^	−0.020675	0.015017	−0.870175	17.067186	2.495747
03.02.2020–16.03.2020	1439	−3.353 × 10^−5^	−0.020675	0.015017	−1.223812	14.9922	2.837796
06.02.2020–19.03.2020	1439	−3.418 × 10^−5^	−0.020675	0.015444	−0.7865	10.45075	2.429242
10.02.2020–23.03.2020	1439	−4.774 × 10^−5^	−0.020675	0.015444	−0.593074	9.164225	2.217087
13.02.2020–26.03.2020	1439	3.318 × 10^−5^	−0.020675	0.015444	−0.481142	7.529997	2.092023
17.02.2020–30.03.2020	1439	2.24 × 10^−5^	−0.020675	0.015444	−0.455545	7.130588	1.905316

**Table A3:** The summary statistics of the intraday log-returns for the dataset associated with the extreme event known as Black Monday due to the COVID-19 pandemic, which occurred on 16 March 2020. The statistics are given for the total period of time (65 working days) and for nine periods (45 working days each) analyzed in terms of the cumulative entropy.

Time Window	Return Count	Mean	Minimum	Maximum	Skewness	Kurtosis	Reference Entropy
Black Monday due to COVID-19 (16.03.2020)							
30.12.2019–30.03.2020	3119	2.629 × 10^−5^	−0.020675	0.015444	−0.41944	14.057644	1.905316
30.12.2019–02.03.2020	2159	2.558 × 10^−5^	−0.01423	0.014709	0.082664	21.582866	1.969378
02.01.2020–05.03.2020	2159	4.673 × 10^−5^	−0.014229	0.014709	0.202652	19.065582	1.40755
06.01.2020–09.03.2020	2159	3.193 × 10^−5^	−0.020675	0.015017	−0.672271	23.334691	2.427632
09.01.2020–12.03.2020	2159	7.846 × 10^−5^	−0.020675	0.015017	−0.83067	21.27912	2.495747
12.01.2020–16.03.2020	2159	−1.302 × 10^−5^	−0.020675	0.015017	−1.320883	20.43049	2.837796
16.01.2020–19.03.2020	2159	−2.282 × 10^−5^	−0.020675	0.015444	−0.856189	14.791115	2.429242
20.01.2020–23.03.2020	2159	1.789 × 10^−5^	−0.020675	0.015444	−0.641751	13.169321	2.217087
23.01.2020–26.03.2020	2159	2.389 × 10^−6^	−0.020675	0.0154434	−0.511374	11.266383	2.092023
27.01.2020–30.03.2020	2159	1.401 × 10^−5^	−0.020675	0.015444	−0.520834	11.015243	1.905316
